# How the COVID-19 pandemic can distort risk adjustment of health plan payment

**DOI:** 10.1007/s10198-021-01346-5

**Published:** 2021-07-15

**Authors:** Richard C. van Kleef, Mieke Reuser

**Affiliations:** 1 Erasmus School of Health Policy & Management, Rotterdam, The Netherlands; 2 Dutch Ministry of Health, Welfare and Sports, The Hague, The Netherlands

**Keywords:** Health insurance, Risk adjustment, COVID-19

## Abstract

The COVID-19 pandemic has led to disruptions in healthcare utilization and spending. While some changes might persist (e.g. substitution of specialist visits by online consultations), others will be transitory (e.g. fewer surgical procedures due to cancellation of treatments). This paper discusses the implications of transitory changes in healthcare utilization and spending for risk adjustment of health plan payment. In practice, risk adjustment methodologies typically consist of two steps: (1) calibration of payment weights for a given set of risk adjusters and (2) calculation of payments to insurers by combining the calibrated weights with enrollee characteristics. In this paper, we first introduce a simple conceptual framework for analyzing the (potential) distortions from the pandemic for both steps and then provide a hypothetical illustration of how these distortions can lead to under- or overpayment of insurers. The size of these under-/overpayments depends on (1) the impact of the pandemic on patterns in utilization and spending, (2) the distribution of risk types across insurers, (3) the extent to which insurers are disproportionately affected by the pandemic, and (4) features of the risk adjustment system.

## Introduction

The COVID-19 pandemic as well as the (lock-down) measures implemented to control the spread of the virus have led to disruptions in healthcare utilization and spending. During the pandemic, utilization of services related to the treatment of COVID-19 such as intensive care and specific medical equipment has been higher than normal. On the other hand, utilization of regular services such as surgical procedures has been lower due to postponement, cancellation or substitution of medical treatments. While some of these changes might persist others are likely to be transitory. This paper focuses on the latter and shows how transitory disruptions in healthcare utilization and spending, even if short-lived, can have implications for risk adjustment of health plan payment for years to come.

Risk adjustment systems provide insurers with payments based on risk characteristics of their enrollees called ‘risk adjusters’. Typical information used as a basis for risk adjuster variables includes age, gender, and diagnoses (from a prior period). Each risk adjuster variable comes with a ‘payment weight’ or ‘risk score’, which is typically higher for the elderly and the chronically ill than for the young and healthy. In practice, risk adjustment procedures roughly consist of two steps: (1) calibration of payment weights for a given set of risk adjusters and (2) calculation of payments to insurers by combining the calibrated weights with enrollee characteristics. This widely accepted approach to risk adjustment and plan payment depends on an assumption that the relationship between the risk adjuster variables and individual spending is reasonably stable between the time weights are calibrated and the time payments are executed. This paper will show how the COVID-19 pandemic disrupts relationships between risk adjusters and spending and thereby complicates both of the aforementioned steps.

Calibration of payment weights (step 1) is generally done by a least-squares regression of healthcare spending on a predefined set of risk adjusters [1]. In most systems, the dataset used for calibration is different from the population and/or year for which risk-adjusted payments to insurers are calculated. For example, the Netherlands uses nationwide spending data from year *t*-3 (in combination with morbidity flags from *t*-4 as well as other risk adjusters) to calibrate weights for payment year *t* [2]. Switzerland uses somewhat more recent spending data, from *t*-1 in combination with morbidity flags from *t*-2 as well as some other risk adjusters [3]. Systems in the United States, such as the Marketplaces (Affordable Care Act) and Medicare Advantage rely on calibration data from both a prior period and a subpopulation [4, 5]. Before running the regression, researchers typically modify the calibration data to make it representative for the payment year. Typical modifications include corrections for cost inflation and for changes in the benefits package and composition of the population. Although there is no doubt that COVID-19 changes patterns in utilization and spending, it is still uncertain what these patterns will eventually look like and how they will differ from those in the past and the future. Because of this uncertainty, it will be harder to make calibration samples representative, both during the pandemic (when the calibration sample comes from the pre-pandemic period) and after the pandemic (when the calibration sample comes from the pandemic period). We will discuss the circumstances under which discrepancies between the calibration sample and the payment year can be problematic.

Calculation of payments to insurers (step 2) is generally done by combining the payment weights from step 1 with characteristics of health plan enrollees. For each enrollee, the combination of risk adjuster flags and payment weights results in a value for ‘predicted spending’. The sum of predicted spending over all individuals enrolled with insurer *k* forms the basis for the payment to *k*. During the pandemic, the postponement, cancellation and/or substitution of care potentially influence patterns in risk adjuster flags. For example, hospitals might have performed fewer surgical procedures (e.g. because of a temporary shortage of intensive care beds or to mitigate the risk of COVID-19 infections among vulnerable patients), resulting in fewer inpatient diagnostic flags. In prospective risk adjustment systems, morbidity flags are based on data from prior years. For a given set of payment weights, fewer procedures in year *t-*1 (e.g. 2020) might lead to lower payments in year *t* (2021). We will discuss and illustrate the circumstances under which such a disruption of risk adjuster flags can be problematic.

In sum, the goal of this paper is to identify and discuss the complications of COVID-19 for risk adjustment of health plan payment. Contrary to many existing papers on the design and evaluation of risk adjustment systems for health plan payment (e.g. [6–10]) our focus will be on under-/overpayment at the level of *insurers*, rather than the level of individual consumers or specific subgroups of the population. Individual- and/or group-level under-/overcompensation are typically used to quantify incentives for insurers to engage in risk selection, i.e. actions to target (deter) consumers that are predictably (un)profitable. Risk selection by insurers, however, is not our primary interest here. Instead, we analyze the circumstances under which COVID-19-related distortions in plan payments can lead to overpayment or underpayment of individual insurers and/or the market as a whole. The underlying idea of our analysis is that over/underpayment at these levels is problematic given that—in most healthcare systems—the impact of COVID-19 on healthcare spending is considered a ‘joint’ responsibility of the regulator, insurers and healthcare providers rather than the responsibility of (individual) insurers alone. Moreover, over/underpayment of individual insurers might affect insurance premiums, which can be considered unfair, both from the viewpoint of insurers and that of consumers.

The structure of this paper is as follows. The following section presents a simple conceptual framework for analyzing how COVID-19 distorts the calibration of payment weights (step 1) and the calculation of payments to insurers (step 2). The subsequent section provides a hypothetical quantitative illustration. The final section summarizes our main findings, discusses the international relevance of these findings, and describes a series of policy measures that could be considered to mitigate the effects of distortions from COVID-19 including (temporary) redesign of current risk adjustment methodology and implementation of risk sharing.

## Conceptual framework

This section introduces a conceptual framework for analyzing the impact of COVID-19 on risk adjustment. More specifically, we will describe how the pandemic potentially disrupts patterns of healthcare utilization and spending (Sect. "How COVID-19 can affect utilization and spending") and then discuss the implications of such disruptions for the calibration of payment weights (Sect. "How the pandemic can complicate calibration of payment weights") and the calculation of payments to insurers (Sect. "How the pandemic can complicate the calculation of payments to insurers").

### How COVID-19 can affect utilization and spending

The (potential) effects of the pandemic on utilization and spending are multidimensional. First, during the pandemic, utilization of services directly related to the treatment of COVID-19 such as intensive care and protective medical equipment is likely to be higher than before and after the pandemic. Second, during high COVID-19 incidence rates, utilization of ‘regular’ services such as inpatient surgery might be lower, at least temporarily, due to lock-down measures and/or allocation of existing hospital beds to COVID-19 patients. Third, in periods with decreased or stabilized COVID-19 incidence rates, utilization of regular treatments might be higher than normal because healthcare providers catch up with some of the postponed treatments. Fourth, for outpatient types of services, in-person doctor visits might be substituted with online consultations. While some of these direct effects on utilization patterns might persist (such as increased use of online consultations) others are likely to be transitory. In sum, these disruptions will occur in the pandemic year and to some degree in years post the pandemic (e.g. due to long COVID or because online consultations for specific purposes remain to be the norm).

In addition, there might be some indirect effects. For example, cancellation and postponement of regular treatments might lead to exacerbations of health problems, which—in the long run—can result in higher spending. At the same time, the cancellation of regular treatments might lead to a decrease in iatrogenic diseases, which might result in lower spending, both in the short and in the long run.^1^

The direction and size of these direct and indirect effects are empirical questions. Given the typical delay in insurance-claim registration and the uncertainty about how the pandemic will develop, it might take years before we know the exact answer to these questions. It is this uncertainty that complicates the calibration of risk adjustment payment weights and the calculation of payments to insurers.

The aforementioned changes in utilization and spending are likely to affect risk adjuster flags in health plan payment systems. For example, a reduction of regular hospital treatments is likely to result in fewer disease flags in morbidity classifications based on diagnoses from hospital settings (such as the Diagnoses-based Cost Groups used in the Netherlands [2], the Hierarchical Morbidity Groups used in Germany [11], the Hierarchical Condition Categories used in the United States [4, 5] and the indicator based on prior hospitalization used in Switzerland [3]). Below, we describe how such effects complicate the calibration of payment weights and the calculation of payments to insurers.

### How the pandemic can complicate calibration of payment weights

Risk adjustment models for health plan payment typically assume a linear relationship between healthcare spending and a predefined set of risk adjuster variables:

1 $${y_{{i,t}}} = {\beta _{0}} + {\beta _{1} x_{{i,1,t}}} + {\beta _{2} x_{{i,2,t}}} + \cdots + {\beta _{p} x_{{i,p,t}}} + {\varepsilon _{{i,t}}} ,$$

where ${y}_{i,t}$ the amount of healthcare spending for individual *i* in year *t*, $x_{{i,j,t}}$ the value for *i* on risk adjuster *j* (typically a dummy variable) in year *t*, $\beta _{0}$ a constant term, $\beta _{j}$ the average effect of risk adjuster *j* on $y$ (holding other risk adjusters fixed) and $\varepsilon _{{i,t}}$ the error term in year *t*. In practice, the number of risk adjuster variables *p* used in risk adjustment models runs into the tens or hundreds. Typical information on which these variables are based includes age, gender, socioeconomic factors and diagnoses (either from year *t* in case of concurrent models or from a prior period in case of prospective models).

Assuming (1) reflects the ‘actual’ relationship between risk adjusters and spending in year *t*, the goal of the regulator is to estimate this relationship as accurately as possible. The best estimation of $\beta _{j}$ can be obtained by a regression of spending on risk adjusters using ***realized*** information from year *t* itself:

2 $$y_{{i,t}} = b_{{0,t}} + b_{{1,t}} x_{{i,1,t}} + b_{{2,t}} x_{{i,2,t}} + \cdots + b_{{p,t}} x_{{i,p,t}} + e_{{i,t}} ,$$

where $x_{{i,j,t}}$ the value for *i* on risk adjuster *j* in year *t* and $b_{j}$ the estimated payment weight for $x_{j}$ in year *t*. This regression inherently provides payment weights that accurately reflect the (assumed linear) relationship between risk adjusters and spending in the payment year. As far as we know, this procedure for estimating payment weights is only applied in Germany [11]. In other countries, the relationship between risk adjusters and spending in payment year *t* is estimated on a calibration sample from a previous period. If we assume a delay *d* (measured in years) between the calibration sample and the payment year, the regression model can be written as

3 $$y_{{i,t - d}} = b_{{0,t - d}} + b_{{1,t - d}} x_{{i,1,t - d}} + b_{{2,t - d}} x_{{i,2,t - d}} + \cdots + b_{{p,t - d}} ,x_{{i,p,t - d}} + e_{{i,t - d}} .$$

In these settings, it is important to make the calibration sample ‘representative’ for payment year *t*. Any discrepancy in the relationship between risk adjusters (*x*) and spending (*y*) between the calibration sample and the population/year of interest can result in payment weights (*b*) that do not accurately reflect the relationship ($\beta$) between risk adjusters and spending in the payment year as presented in (1). Typical modifications of calibration samples to avoid such discrepancies include corrections for cost inflation and for changes in the benefits package and composition of the population. Until recently, in most settings, (modified) calibration samples have been considered reasonably representative. Due to COVID-19, however, this might no longer be the case, at least not for the years to come. The direction and size of discrepancies between the calibration sample and the payment year are uncertain (as long as accurate data on the impact of COVID-19 on spending is lacking) and might differ from setting to setting.

### How the pandemic can complicate the calculation of payments to insurers

In the second step of risk adjustment, the estimated payment weights are combined with enrollee characteristics to generate a prediction of spending for insurer *k* that forms the basis for the payment to *k* in year *t*. Both here and in our quantitative illustration we assume $\hat{y}_{{k,t}}$ is calculated as

4 $$\hat{y}_{{k,t}} = \mathop \sum \limits_{{i \in k}} \left( {b_{{0,t - d}} + b_{{1,t - d}} x_{{i,1,t}} + b_{{2,t - d}} x_{{i,2,t}} + \cdots + b_{{p,t - d}} x_{{i,p,t}} } \right),$$

which is the sum of predicted spending over all individuals enrolled with insurer *k*. It is straightforward to see how ‘biased’ payment weights *b* from (3) would result in a ‘biased’ value of predicted spending, an argument already made in the previous section. For example, if $b_{{0,~t - d}}$ resulting from (3) is higher (lower) than $b_{0}$ in (1) then predicted spending for insurer *k* might be too high (low), ceteris paribus.

Apart from the issue of distorted weights, biased payments can also result from disruptions in patterns of risk adjuster flags (*x*) directly. In several health plan payment systems, risk adjuster flags are based on information from a prior period. Such risk adjusters are commonly referred to as ‘prospective’ risk adjusters, as opposed to ‘concurrent’ risk adjusters that are based on information from the payment year itself. Prospective risk adjusters are used in Germany (where Hierarchical Morbidity Groups are based on diagnoses from the prior year [11]) and Switzerland (where Pharmaceutical Cost Groups and an indicator for yes/no hospitalization are based on information from the prior year [3]). The Netherlands goes even further using information from up to *five* consecutive prior years as a basis for spending-based risk adjusters [2].^2^ With prospective risk adjusters, a disruption in patterns of utilization can influence payments in future years, *even when the payment weights b are ‘unbiased’*. The next section provides a hypothetical illustration of these dynamics.

Examples of concurrent risk adjusters can be found in the US under Medicare Advantage and the Affordable Care Act [4]. With concurrent risk adjusters, irregularities in flags (*x*) might be less of a problem than with prospective risk adjusters since flags in year *t* are directly related to spending in year *t*. For example, a reduction in hospital treatments in *t* is likely to result in both fewer diagnostic flags and lower spending. This implies that for insurers with fewer diagnostic flags, both the risk-adjusted payment and spending are likely to reduce. With prospective risk adjustment, on the other hand, fewer flags based on information from year *t*-1 result in a reduction of the payment for year *t*, but not (necessarily) in a reduction of spending in year *t*, creating a gap between payments and spending.

### Key observations

In sum, we can conclude that disruptions in healthcare utilization and spending due to the COVID-19 pandemic can distort health plan payments, either via the estimation model (i.e. the regression model used to estimate payment weights) or via the payment model (i.e. the formula used to calculate payments to insurers). These distortions potentially result in under-/overpayment of individual insurers and threaten the level playing field. Moreover, in systems where payments to plans are ‘fixed’ ex ante—such as in the Netherlands [2]—under-/overpayment might also occur at the market level. In systems where payments are designed to sum to zero (ex post)—such as in Switzerland [3] and in the US Marketplaces [4]—under-/overpayment might occur at the insurer level, but not at the market level.

## A hypothetical illustration

This section provides a hypothetical quantitative illustration of how the pandemic can distort the estimation of payments weights (step 1) and calculation of payments to insurers (step 2). To do so, we make some assumptions on how COVID-19 interferes with the regular pattern of risk adjuster flags and how this affects the estimation model and the payment model. For this exercise, we assume that payments to plans are fixed ex ante (which implies that under-/overpayments can occur both at the level of individual insurers and at the market level). More specifically, we assume that payments simply equal predicted spending according to formula (4). It should be emphasized that our simulation is purely hypothetical; it is not our intention to replicate either the Swiss risk adjustment system or any other real-world system. In practice, outcomes can be (very) different depending on contextual factors (such as the scope and depth of coverage), the specific risk adjustment methodology in place and the actual impact of the pandemic on spending and risk adjuster flags. The goal of our illustration is to give an impression of how and under what circumstances COVID-19 can distort payments to insurers.

The data available for our simulation comes from a Swiss insurance company and contain information on individual-level spending and risk adjuster flags for about 1.3 million individuals. Spending is presented in Swiss francs (CHF)^3^ and comprises all insurance claims for services covered by the Swiss basic health insurance in 2016. Risk adjuster flags include age, gender, prior hospitalization and pharmaceutical-based cost groups (PCGs). For the purpose of our illustration, we primarily focus on ‘prior hospitalization’.^4^ This risk adjuster takes the form of a dummy variable indicating whether a person spent at least three consecutive nights in a hospital or nursing home in 2015. Combined with spending in 2016, this risk adjuster allows us to run a series of simulations for a simple prospective risk adjustment model. In a later step, we extend this risk adjustment model with the risk adjuster ‘yes/no PCG’ to show how a distortion of one risk adjuster can be mitigated by the presence of another. PCGs in our dataset are derived from the use of specific prescribed drugs in 2015. Although the PCG classification distinguishes multiple diseases, we restrict our analysis to ‘yes/no PCG’, again for simplification.

Table 1 presents some key statistics that provide a starting point for our simulation. Mean per person spending in the population equals CHF 3,844. For the purpose of our analysis, per person spending is annualized by dividing observed spending by the fraction of the year an individual was enrolled, a common procedure in risk adjustment. All figures presented in this section are weighted by this fraction.

**Table 1:** Descriptive statistics

	Frequency in year t		Mean spending year t (CHF)	% prior hosp.		% PCG	
	Insured years	% Of total population		No	Yes	No	Yes
Descriptive statistics							
Total population	1,255,321	100	3,844				
Prior hospitalization = no	1,187,026	94.6	3,177			81.0	19.0
Prior hospitalization = yes	68,295	5.4	15,431			38.8	61.2
PCG = no	988,414	78.7	2,059	97.3	2.7		
PCG = yes	266,908	21.3	10,453	84.3	15.7		
Insurers with roughly identical portfolios							
Insurer 1	627,716	50.0	3,843	94.6	5.4	78.7	21.3
Insurer 2	627,605	50.0	3,845	94.6	5.4	78.7	21.3
Insurers with non-identical portfolios							
Insurer 1	627,766	50.0	4,239	91.3	8.7	77.4	22.6
Insurer 2	627,556	50.0	3,448	97.8	2.2	80.1	19.9

1 CHF = 0.91 euro = 1.11 U.S. dollar (on May 19, 2021)

Table 1 also shows the mean per person spending in two mutually exclusive groups based on whether an individual is flagged by the risk adjuster ‘prior hospitalization’. Relative to the total population, 5.4% is flagged by this risk adjuster with mean per person spending of CHF 15,431. For the complementary group (94.6%) mean per person spending equals CHF 3,177. The difference in mean spending between these groups indicates that prior hospitalization is indeed predictive of spending in year *t*. A similar observation can be made for the risk adjuster ‘PCG = yes’: for the group of enrollees flagged by this risk adjuster (21.3% of the population) mean spending equals CHF 10,453 per person per year; for the complementary group (78.7%) mean spending equals CHF 2,059 per person per year.

In terms of payment fit, we are primarily interested in the under-/overpayment of insurers (see Introduction). As will be described in more detail below, we simulate under-/overpayment for two hypothetical insurers that each cover 50% of the population. Two market scenarios are taken into account. In the first scenario, insurers have roughly identical portfolios. For this scenario, we randomly assign 50% of the population to insurer 1 and the other 50% to insurer 2. In the second scenario, insurer 1 has a disproportionate share of high-risk people while insurer 2 has a disproportionate share of low-risk people. For this scenario, we randomly assign 80% of the group with ‘prior hospitalization = yes’, together with a random 48.3% of the group with ‘prior hospitalization = no’ to insurer 1. All individuals not assigned to insurer 1 (i.e. 20% of the group with ‘prior hospitalization = yes’ and 51.7% of the group ‘prior hospitalization = no’) are assigned to insurer 2. Table 1 presents the portfolio size and mean spending for the two insurers per scenario. The third and fourth columns to the right show that with non-identical portfolios insurer 1 indeed has more enrollees with ‘prior hospitalization = yes’ than insurer 2, resulting in a difference in mean per person spending of about 800 CHF. With non-identical portfolios, insurer 1 also has a disproportionate share of people with ‘PCG = yes’.

### Distortion of the calibration sample

As a first step in our illustration, we show how biased payment weights can affect outcomes for individual insurers. This illustration can be framed as follows. Assume that spending and risk adjuster flags in our dataset are perfectly representative of the payment year. In the calibration step, the objective of the regulator is to find the ‘right’ payment weights to be used in formula (4). To estimate these weights, the regulator relies on a calibration sample from a previous period. We simulate three scenarios regarding this calibration sample. For each scenario we estimate payment weights for a risk adjustment model with two dummy variables (and no intercept): one dummy for ‘prior hospitalization = yes’ and one for ‘prior hospitalization = no’. The model takes the form of a least-squares regression of (annualized) spending on these two dummy’s (with a weight for duration of enrollment).

The first scenario (W0, with ‘W’ for weights) assumes that the calibration sample is *not* distorted by the pandemic. More specifically, we assume there is no discrepancy between the calibration sample and the payment year. As shown in Table 2, the payment weights in this scenario simply equal the mean per person spending for the respective groups that were already presented in Table 1. As indicated by the *R*-squared, the risk adjustment model explains 6.9% of the variance in spending in our dataset.

**Table 2:** Payment weights in three scenarios regarding the calibration sample

	Frequency of flags in calibration sample (%)	Payment weight (CHF)
Scenario W0: no distortion of calibration sample		
≥ 3 nights hospitalized in *t*−1 = no	94.6	3,177
≥ 3 nights hospitalized in *t*−1 = yes	5.4	15,431
*R*-squared	0.069	
Scenario W1: random distortion of calibration sample		
≥ 3 nights hospitalized in *t*−1 = no	95.1	3,246
≥ 3 nights hospitalized in t−1 = yes	4.9	15,456
*R*-squared	0.068	
Scenario W2: non-random distortion of calibration sample		
≥ 3 nights hospitalized in *t*−1 = no	95.1	3,192
≥ 3 nights hospitalized in *t*−1 = yes	4.9	16,559
*R*-squared	0.068	

The second scenario (W1) assumes the calibration sample is distorted by the pandemic in the following way: flags for ‘prior hospitalization = yes’ decreased by a random 10%. For this scenario, we randomly assign 10% of the group with ‘prior hospitalization = yes’ to the group with ‘prior hospitalization = no’, which is reflected in the frequency of these flags. Because of this distortion, the group with no prior hospitalization becomes somewhat more heterogeneous, resulting in an increase of the payment weight for this group and a slight reduction in the *R*-squared of the estimation model (see Table 2).

The third scenario (W2) contains another type of distortion: like in W1, flags for prior hospitalization in the calibration sample decreased by 10%, but this time the drop is non-random. More specifically, the decrease in flags took place among the relatively low-risk people within the group ‘prior hospitalization = yes’. To make a distinction between the relatively low risks and relatively high risks in the group ‘prior hospitalization = yes’ we calculated median spending in this group in 2015. Those with below-median spending were labeled as relatively low-risk people. For a random 20% of these low-risk people, we changed the flag ‘prior hospitalization = yes’ to ‘prior hospitalization = no’. As shown in Table 2, this results in a decrease in flags for prior hospitalization from 5.4 to 4.9%, similar to scenario W1. Contrary to W1, however, this drop makes the group with ‘prior hospitalization = yes’ more homogenous, which is reflected in a higher weight for this group compared to W0 and W1. In scenario W2, the group ‘prior hospitalization = no’ is more heterogeneous than in W0, but less heterogeneous than in W1, resulting in a payment weight in between the corresponding weights in W0 and W1.

The main takeaway from Table 2 is that estimated payment weights are driven by the composition of the calibration sample (in terms of spending and risk adjuster flags). Our primary question for what follows is how the differences in payment weights in Table 2 affect outcomes for individual insurers. To simulate these outcomes we plug in the estimated payment weights in formula (4) while keeping ‘risk adjuster flags’ constant (that is, we use the flags as present in our original dataset).

**Fig. 1:**
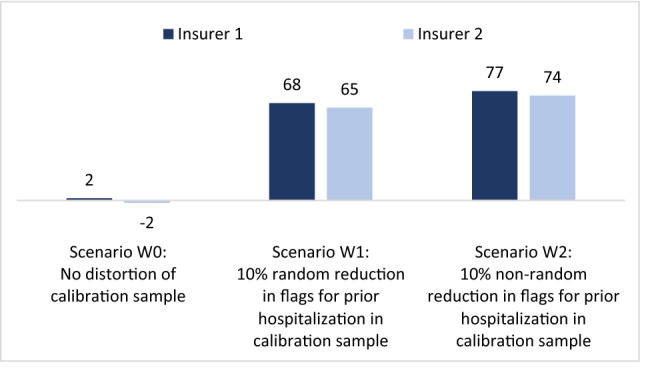
Mean financial result in CHF per person per year for insurers with *roughly identical* portfolios Both insurers cover a random 50% of the population. See ‘roughly identical insurers’ in Table 1

Figure 1 shows the mean financial result under scenarios W0–W2 for two insurers with roughly identical portfolios. The mean financial result is calculated as the mean per person risk-adjusted payment minus the mean per person actual spending in our dataset. Three important observations can be made. First, without a distortion (scenario W0, in which the calibration sample is perfectly representative for the payment year), the financial result for both insurers equals nearly zero. Second, with a random distortion (scenario W1, in which the calibration sample includes a random 10% decrease in flags for ‘prior hospitalization = yes’), both insurers are confronted with a positive financial result. The explanation is that—due to the distortion in the calibration sample—the payment weight for the flag ‘prior hospitalization = no’ is too high. Third, with a non-random distortion (scenario W2, in which the calibration sample includes a non-random decrease in flags for ‘prior hospitalization = yes’), the overpayment for both insurers is even larger than in W1. The reason is that in W2, both the payment weight for ‘prior hospitalization = yes’ and ‘prior hospitalization = no’ are too high. Despite the average overpayment in the market, the distortions in the calibration sample do not alter the level playing field: in all three scenarios, the overpayment for the two insurers is more or less similar.

**Fig. 2:**
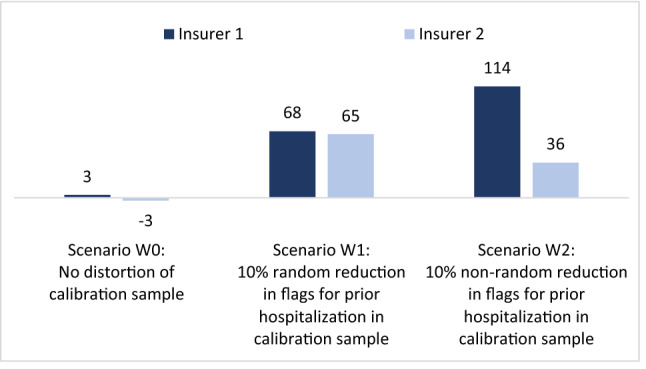
Mean financial result in CHF per person per year for insurers with *non-identical* portfolios. Both insurers cover 50% of the population. Insurer 1 covers more people with prior hospitalization than insurer 2. See rows for ‘non-identical insurers’ in Table 1

Like Fig. 1, Fig. 2 presents the mean financial result under scenarios W0–W2. This time, however, the insurers are assumed to have non-identical portfolios. More specifically, insurer 1 covers more people with prior hospitalization than insurer 2. Again three observations can be made. First, without a distortion (scenario W0), the mean financial result is nearly zero for both insurers, despite the substantial differences in mean spending between the two portfolios (see rows for ‘non-identical insurers’ in Table 1). The explanation is that the risk adjustment model perfectly compensates insurer 1 (insurer 2) for the overrepresentation (underrepresentation) of people with prior hospitalization. Second, with a random distortion (scenario W1, in which the calibration sample includes a random 10% decrease in flags for ‘hospitalization = yes’), both insurers are confronted with a positive financial result. Again, the explanation is that—due to the distortion in the calibration sample—the payment weight for the flag ‘prior hospitalization = no’ is too high. Both insurers benefit from this since both cover many people with ‘prior hospitalization = no’ (prevalence of this flag is 91% for insurer 1 and 98% for insurer 2).^5^ The most remarkable outcome in Fig. 2 is for scenario W2. In this scenario (where the calibration sample includes a non-random decrease in flags for ‘prior hospitalization = yes’), the overpayment for insurer 1 is substantially higher than for insurer 2. The reason is to be found in the higher prevalence of ‘prior hospitalization = yes’ for insurer 1 (8.7%) compared to insurer 2 (2.2%). Due to this difference, insurer 1 benefits more from the ‘biased’ payment weight for this risk adjuster than insurer 2.^6^

In sum, the illustration above leads us to two important conclusions. First, biased payment weights resulting from distortions in the calibration sample can result in overpayment or underpayment of the entire market.^7^ (Although our scenarios W1 and W2 result in overpayment, distortions can go in the other direction as well. This might be the case, for instance, when disease flags increase instead of decrease, e.g. due to an increase of COVID-19-related treatments.) Second, to the extent that insurers’ portfolios differ in terms of composition, biased payment weights can distort the level playing field.

### Distortion of flags used in the payment model

As a second step in our quantitative illustration, we show how a distortion of ‘risk adjuster flags’ can affect outcomes for individual insurers through the payment model. This illustration can be framed as follows. Assume that payment weights are perfectly representative of the relationship between risk adjusters and spending in the payment year. In other words, we assume that formula (4) includes the ‘right’ set of payment weights $b$. Our focus here is on the set of risk adjuster flags ($x$) for which we simulate three scenarios. These scenarios differ in terms of the risk adjuster flags ($x$) used for calculating payments while keeping payment weights ($b$) constant. The first scenario (F0, with ‘F’ for flags) assumes that the risk adjuster flags used for the calculation of plan payments are *not* distorted by the pandemic. In this scenario, we use the flags as present in our dataset. The second scenario (F1) assumes a random distortion of flags used for the calculation of plan payments: flags for prior hospitalization decreased by a random 10%. For this scenario, we randomly assign 10% of the group with ‘prior hospitalization = yes’ to the group with ‘prior hospitalization = no’. The third scenario (F2) assumes a non-random distortion of flags used for calculation of payments: like in scenario F1, flags for prior hospitalization decreased by 10%, but this time the drop is non-random. More specifically, the decrease took place in the portfolio of *one* insurer (for which the number of flags for ‘prior hospitalization = yes’ decreased by 20%). In all three scenarios, the payment weight for ‘prior hospitalization = no’ equals 3,177 CHF and the weight for ‘prior hospitalization = yes’ equals 15,431 CHF (i.e. the undistorted weights under scenario W0 in Table 2).

In what follows, we are interested in how disruptions in risk adjuster flags distort payments to insurers. Similar to the procedure used in the previous section we calculate under-/overpayments for insurers as the mean per person risk-adjusted payment minus the mean per person spending. Although (in F1 and F2) risk adjuster flags for yes/no hospitalization in year *t*-1 are disrupted, we assume that spending in year *t* is not. Again, we run our simulations for roughly identical portfolios and non-identical portfolios.

**Fig. 3:**
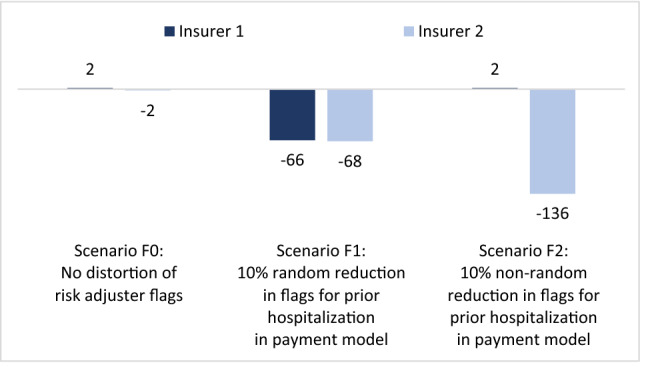
Mean financial result in CHF per person per year for insurers with *roughly identical* portfolios. Both insurers cover a random 50% of the population. See ‘roughly identical insurers’ in Table 1

Figure 3 shows the outcomes of our simulation for two roughly identical portfolios. As expected, the mean financial result for insurers in scenario F0 (no distortion in risk adjuster flags) equals nearly zero. Slight deviations from zero are due to random variation. A decrease in flags for ‘prior hospitalization = yes’ (scenarios F1 and F2) results in lower payments to insurers. When levels of actual spending remain the same (as we assume here) both insurers are confronted with an underpayment. Not surprisingly, a decrease in flags for ‘prior hospitalization = yes’ hits some insurers harder than others as this decrease is concentrated in specific plans (scenario F2). To some extent, scenario F2 might occur when insurers have relatively high market shares in regions that have seen relatively high COVID-19 incidence rates.

**Fig. 4:**
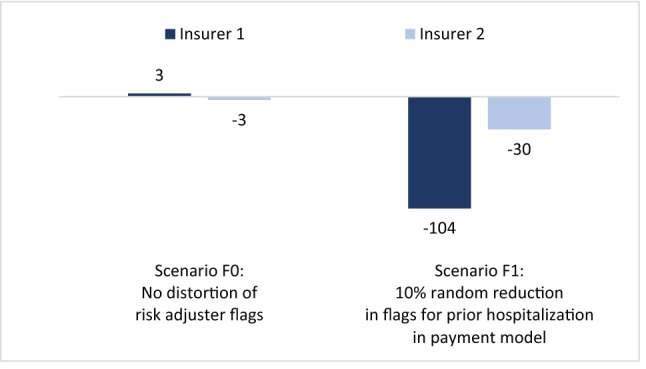
Mean financial result in CHF per person per year for two insurers with ***non-identical*** portfolios. Both insurers cover 50% of the population. Insurer 1 covers more people with prior hospitalization than insurer 2. See rows for ‘non-identical insurers’ in Table 1

Figure 4 shows the outcomes of our simulation for two *non-identical* portfolios. Again, as expected, the mean financial result for insurers in scenario F0 (no distortion in risk adjuster flags in the payment model) equals nearly zero, implying that the risk adjustment model fully compensates for the difference in mean spending between the two insurers. Similar to Fig. 3, a decrease in flags for ‘prior hospitalization = yes’ (scenarios F1) results in lower payments to insurers. This time, however, the effects for insurers are different. Although the mean underpayment in the market is similar to that in Fig. 3, insurer 1 is now losing more money than insurer 2. The explanation is that insurer 1 has more enrollees flagged by the risk adjuster ‘prior hospitalization = yes’ (8.7% of his portfolio) than insurer 2 (2.2%). Consequently, the reduction in flags in the payment model hurts insurer 1 more than insurer 2.

For simplicity, Fig. 4 does not include F2. It is easy to see how a non-random decrease in flags for ‘prior hospitalization = yes’ would change the results compared to scenario F1: if flags would decrease for one insurer but not for the other, the underpayment would be concentrated with that insurer (see Fig. 3), irrespective of whether insurers have identical portfolios or not.

In sum, Figs. 3 and 4 lead us to two important conclusions. First, distortions in risk adjuster flags in the payment model can result in underpayment or overpayment of the entire market.^8^ (Although our scenarios F1 and F2 result in underpayment, distortions can go in the other direction as well, e.g. when disease flags increase instead of decrease as a result of more COVID-19 related treatments.) Second, to the extent that distortions in risk adjuster flags are more severe for some insurers than for others (e.g. due to non-identical portfolios and/or relatively high COVID-19 incidence rates in regions with concentration of specific insurers), disruptions in risk adjuster flags can alter the level playing field.

### How other risk adjusters might rescue the risk adjustment system

So far, our simulations assumed that the risk adjustment system includes just one risk adjuster, i.e. ‘yes/no prior hospitalization’. In practice, risk adjustment systems are much more sophisticated and often include a wide range of risk adjusters. An interesting question is how the outcomes of our simulations would change if we included additional risk adjusters. To answer this question we repeated our simulation for a risk adjustment system that includes both ‘yes/no prior hospitalization’ and ‘yes/no PCG’. Table 3 summarizes the outcomes of this simulation and compares these outcomes with those of our previous simulation. More specifically, Table 3 shows the overall mean per person financial result (which indicates the mean per person under-/overpayment across the entire market) and the absolute difference in mean per person financial result between insurer 1 and insurer 2 (which indicates the extent to which the level playing field is being altered). The results in the columns ‘Without PCG’ replicate the outcomes of our previous simulation with only one risk adjuster. The columns ‘With PCG’ show the outcomes for the risk adjustment system with two risk adjusters; in all scenarios, we assume that flags for the risk adjuster ‘yes/no PCG’ are not distorted by the COVID-19 pandemic.

**Table 3:** Outcomes per scenario without/with risk adjuster ‘PCG’ added to the risk adjustment system

Scenario	Overall mean per person financial result across the market (CHF)		Absolute difference in mean per person financial result between insurer 1 and 2 (CHF)	
	Without PCG	With PCG	Without PCG	With PCG
Roughly identical insurers				
W0	0	0	3	4
W1	67	50	3	4
W2	75	58	3	4
Non-identical insurers				
W0	0	0	6	7
W1	67	50	3	6
W2	75	58	78	72
Roughly identical insurers				
F0	0	0	3	4
F1	− 67	− 50	1	2
F2	− 67	− 50	137	105
Non-identical insurers				
F0	0	0	6	7
F1	− 67	− 50	73	53

Results in columns ‘Without PCG’ can be derived from Figs. 1, 2, 3, 4. For example, Fig. 1 indicates that in scenario W0 the mean per person financial result (overall) equals zero and that the absolute difference in mean per person financial result between the two insurers equals 4 euro. Small differences are due to rounding.

Figures 1, 2, 3 and 4 have shown that scenarios W1, W2, F1 and F2 result in under- or overpayment across the market (see also column 2 of Table 3). Column 3 of Table 3 shows that these under-/overpayments shrink when the risk adjuster ‘PCG’ is added. A similar observation can be made for the absolute difference in mean per person financial result between the insurers: in the scenarios ‘Non-identical insurers: W2’, ‘Roughly identical insurers: F2’ and ‘Non-identical insurers: F1’ this difference is substantially smaller in the risk adjustment system ‘With PCG’ (column 5) than in the system ‘Without PCG’ (column 4). In other words, the distortions of both the estimation model and the payment model are smaller for a system with both ‘yes/no prior hospitalization’ and ‘yes/no PCG’ than for a system with only the former. The explanation can be found in Table 1 (in the four columns to the right): people with prior hospitalization have a relatively high probability of being flagged by a PCG, and vice versa. The correlation between the two risk adjusters in our dataset turns out to be 0.237. Due to this correlation, adding ‘yes/no PCG’ as a risk adjuster reduces the payment weight for the risk adjuster ‘yes/no prior hospitalization’ implying that a distortion of this risk adjuster results in less damage. This brings us to an interesting observation: distortions from COVID-19 via a specific risk adjuster, say $x_{1}$ (e.g. ‘yes/no prior hospitalization’), are smaller as the risk adjustment system includes additional risk adjusters that are correlated with $x_{1}$ AND are not (substantially) affected by the pandemic. The extent to which ‘other risk adjusters can rescue a risk adjustment system’ is an empirical question depending on the features of that system and the extent to which these other risk adjusters positively correlate with those distorted by COVID-19.

## Discussion

Our analysis has shown how the COVID-19 pandemic can distort risk adjustment systems. Through disruptions in spending and risk adjuster flags, the pandemic can distort both the estimation model and the payment model. Our hypothetical illustration has shown that these distortions can lead to under-/overpayment of insurers. The relevance of the illustration is to be found in the ‘direction’ in which distortions work rather than the ‘size’ of under-/overpayment. How distortions actually work out in a specific setting depends on (1) the impact of COVID-19 on patterns in utilization and spending, (2) the distribution of risk types across insurers, (3) the extent to which insurers are unequally affected by the pandemic and (4) specific features of the risk adjustment methodology in place.

At the time of revising this paper for the European Journal of Health Economics, some first estimations have become available about the impact of COVID-19 on spending and utilization. In the Netherlands, a decrease of 4.7% has been estimated for total healthcare spending in 2020 relative to pre-pandemic years; for hospital care (non-COVID-19), the estimated decrease in 2020 equals 8.9% [13]. In Switzerland, an increase of 0.4% in total healthcare spending has been estimated for 2020 compared to 4% in earlier years [14]. These estimations indicate that patterns in spending and utilization are indeed disrupted by the pandemic, potentially resulting in the type of distortions discussed in this paper.

The international relevance of our findings is threefold. First, any system relying on a calibration sample from a previous period will be confronted with a potential distortion of estimated payment weights due to discrepancies between the calibration sample and the payment year. For example, this is true for the risk adjustment systems in The Netherlands, Switzerland and specific sectors in the United States such as Medicare Advantage and the Marketplaces under the Affordable Care Act. Second, in any system with prospective risk adjusters, discontinuities in flags might complicate the payment model since these discontinuities (e.g. fewer diagnostic flags based on data from year *t*-1) do not necessarily follow spending patterns (in year *t*). Prospective risk adjusters are used in Germany, The Netherlands and Switzerland. Third, distortions from COVID-19 via a specific risk adjuster, say ${x}_{1}$ (e.g. ‘yes/no prior hospitalization’), are smaller as the risk adjustment system includes additional risk adjuster variables that are positively correlated with ${x}_{1}$ *and* that are not affected by the COVID-19 pandemic. In this respect, distortions might be bigger in systems that heavily rely on hospital information as a basis for morbidity-adjusters than systems that use information from other healthcare settings as well.

How distortions actually translate to under-/overpayment depends on how payments are calculated. Where payments to plans are fixed ex ante—an approach assumed in our hypothetical example and common practice in the Netherlands—under-/overpayment can occur both at the level of individual insurers and at the level of the entire system. Where payments are designed to sum to zero (ex post)—such as in the Swiss basic health insurance [3] and in the US Marketplaces [4]—under-/overpayment might occur at the insurer level, but not at the market level. When it comes to the effects of distortions on under-/overpayment it is also important to emphasize that the analysis in this paper is limited to one year (or one contract period). Under specific circumstances, it might be possible that under(over)payment for insurer *k* this year will be followed by an over(under)payment for *k* in the next year. For example, a reduction in regular hospital treatments for *k* this year could result in (ex post) overcompensation for *k* this year (since actual spending turns out to be lower than predicted spending) and an undercompensation next year (because of a temporary reduction in prospective morbidity flags).

Regulators could consider two general options to mitigate (the effects of) the impact of COVID-19 on risk adjustment: (1) redesign of risk adjustment methodology and/or (2) supplementary risk-sharing measures. An example of the first option is to calibrate payment weights in retrospect using realized spending and risk adjuster flags. This methodology, which is the standard procedure in Germany, makes sure that payment weights accurately reflect actual relationships between spending and risk adjusters in the year of interest (and thus avoids issues related to discrepancies between the calibration sample and year of interest). Instead of a full retrospective estimation model, regulators could consider modifying payment weights only for risk adjusters that are potentially problematic. Another example is to switch from prospective to concurrent risk adjusters. Concurrent models, as applied in risk adjustment systems under the Affordable Care Act and in Medicare Advantage, might mitigate under-/overpayment due to distortions in risk adjuster flags since these distortions are likely to go hand in hand with distortions in spending. In a concurrent system, a reduction in diagnoses, for instance, is likely to result in both fewer diagnostic flags and (thus lower values of predicted spending) and lower values of actual spending. In a prospective system changes in payments and spending are less likely to go hand in hand. A third example of risk adjustment redesign is to simply ‘keep out’ problematic years, e.g. by not using calibration samples with crucial information (i.e. spending and/or risk adjuster flags) from 2020.

An example of the second option is risk sharing between insurers and the regulator [15, 16]. A possible measure could be, for instance, that the regulator subsidizes the market in case of an overall underpayment and ‘takes back’ money in case of an overall overpayment. Subsidies and repayments could be calculated in proportion to the initial risk-adjusted payment. Such a form of risk sharing is implemented in the Netherlands for 2021 to protect both the regulator and the market against a mismatch between payments and spending. Another modality is to apply a risk corridor for individual insurers in a way that they are subsidized for losses greater than X and/or cut for profits greater than Y. This modality can be effective in protecting individual insurers from excessive financial risk and might mitigate distortions of the level playing field. Risk corridors can be applied either between insurers and the regulator (which implicitly protects the regulator and the market for excessive under-/overpayment) or among insurers (leaving market-level under-/overpayments intact). A third risk sharing option is to ‘carve out’ specific types of spending that are likely to be distorted. Other forms of risk sharing such as outlier-risk sharing might be less effective tools to deal with the impact of COVID-19.

Another potentially interesting form of risk sharing is between insurers and providers. To the extent that the pandemic reduces spending on regular types of care, surpluses on the side of insurers might help to compensate losses on the side of providers and vice versa. Such risk sharing can be organized by the regulator or the market itself. In The Netherlands, for instance, insurers and providers themselves agreed to move funds from insurers to providers in order to prevent providers from going bankrupt. The occurrence of COVID-19 might lead societies to reconsider the risks associated with a pandemic: should such risks be borne by insurers and/or healthcare providers or should it be borne by the regulator?

As a final remark, we would like to emphasize that the relevance of this paper is not ‘limited’ to the case of COVID-19. In fact, the conceptual analysis and hypothetical illustration can be useful to understand the potential effects of any disruptive change in healthcare spending and utilization on risk adjustment.

## Footnotes

1 For a helpful description of the potential long-term implications of COVID-19, see https://hcldr.wordpress.com/2020/04/07/the-pandemics-4th-wave/.

2 An argument for using prospective instead of concurrent risk adjusters is that the latter might not only correct for predictable spending variation but also for *un*predictable spending variation [12].

3 1 CHF = 0.91 euro = 1.11 U.S. dollar on May 19, 2021.

4 As we will show later on, the main point of our illustration is that a disruption of flags regarding the risk adjuster ‘prior hospitalization’ (either in the calibration sample or in the payment year itself) can distort payments to insurers. For making this point, it is not necessary to include other risk adjusters like age and gender.

5 Surprisingly, the effect is more or less the same for insurers 1 and 2 despite the 7 percent point difference (98%–91%) in prevalence of ‘prior hospitalization = no’. The explanation for this counterintuitive effect is that the payment weight for ‘prior hospitalization = yes’ also increases somewhat compared to scenario W0 (see Table 2; caused by random variation) which mitigates the net effect. A second explanation is that in relative terms the difference in prevalence of ‘prior hospitalization = no’ between the two insurers is rather small.

6 Note that both insurers also benefit from the ‘biased’ payment weight for ‘prior hospitalization = no’.

7 Note that our simulation assumes that payments are fixed ex ante, i.e. they simply equal predicted spending as in formula (4). In systems where payments are designed to sum to zero (ex post)—such as in Switzerland [3] and in the U.S. Marketplaces [4]—under/overpayment can occur at the insurer level, but not at the market level.

8 Note that our simulation assumes that payments are fixed an ex ante, i.e. they simply equal predicted spending as in formula (4). In systems where payments are designed to sum to zero (ex post)—such as in Switzerland [3] and in the U.S. Marketplaces [4]—under/overpayment can occur at the insurer level, but not at the market level.
